# Vulnerabilities of Live-Streaming Services in Korea

**DOI:** 10.3390/s22103766

**Published:** 2022-05-15

**Authors:** Sun-Hong Hwang, Ga-Yeong Kim, Su-Hwan Myeong, Tai-Sic Yun, Seung-Min Yoon, Tai-Ho Kim, Ieck-Chae Euom

**Affiliations:** 1 System Security Research Center, Chonnam National University, Gwangju 61186, Korea; shhwang@nshc.net (S.-H.H.); detaimer@jnu.ac.kr (G.-Y.K.); 2 Computer & Telecommunications Engineering, Mirae Campus, Yonsei University, Wonju 26493, Gangwon-do, Korea; msh8206@yonsei.ac.kr; 3 Department of Cyber Defense, Korea University, Seoul 02841, Korea; yts3097@korea.ac.kr (T.-S.Y.); smyoon99@korea.ac.kr (S.-M.Y.); 4 ALL IT ONE Inc., Seoul 08390, Korea; kimtaiho5412@naver.com; 5 Department of Data Science, Chonnam National University, Gwangju 61186, Korea

**Keywords:** vulnerability analysis, grid computing, live-streaming service, threat modeling, STRIDE

## Abstract

Recently, the number of users and the demand for live-streaming services have increased. This has exponentially increased the traffic to such services, and live-streaming service platforms in Korea use a grid computing system that distributes traffic to users and reduces traffic loads. However, ensuring security with a grid computing system is difficult because the system exchanges general user traffic in a peer-to-peer (P2P) manner instead of receiving data from an authenticated server. Therefore, in this study, to explore the vulnerabilities of a grid computing system, we investigated a vulnerability discovery framework that involves a three-step analysis process and eight detailed activities. Four types of zero-day vulnerabilities, namely video stealing, information disclosure, denial of service, and remote code execution, were derived by analyzing a live-streaming platform in Korea, as a representative service, using grid computing.

## 1. Introduction

The fourth industrial revolution has significantly increased activity in the IT industry. As the use of PCs, smartphones, IoT, etc., increases, the software market continues to evolve. However, in the past three years, the number of vulnerabilities in systems (i.e., applications, services, mobiles, IoT, content management systems (CMSs), and ActiveX) has also increased. The number of infringement incidents that exploit software vulnerabilities is increasing with the development of the current software market. To prevent this, we analyzed the exploitation of potential vulnerabilities from an attacker’s perspective. Preemptively removing these is necessary for the industry to progress.

**Figure 1:**
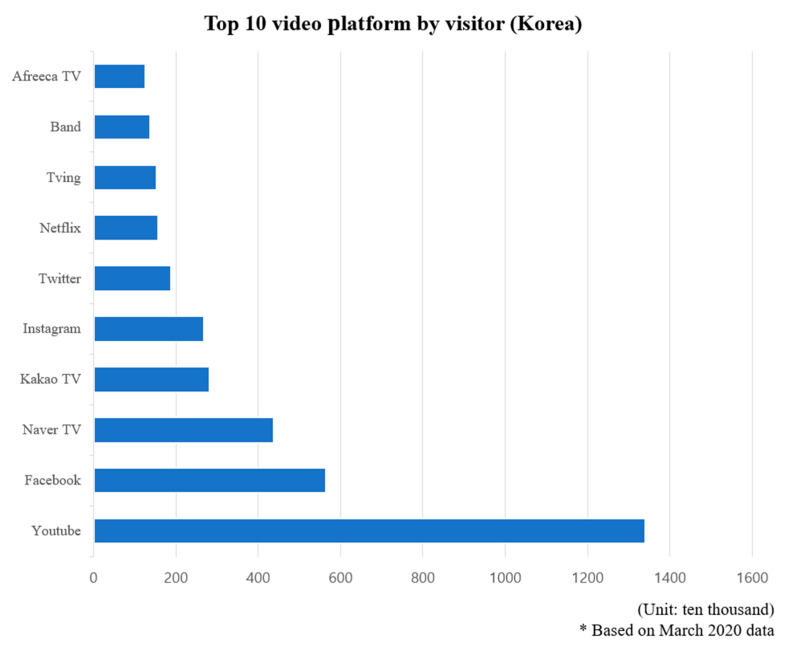
Top 10 video platforms in terms of the number of visitors in Korea.

Social distancing has been implemented because of COVID-19, and live-streaming services are often used for non-face-to-face events, classes, and conferences by organizations such as schools and companies. As shown in Figure 1, the most used live-streaming platforms in Korea are YouTube and Facebook, which have many users worldwide. However, Naver TV, Kakao TV, and Afreeca TV, developed by Korean companies, also have many users in Korea [1]. These live-streaming platforms use grid computing technology to provide high-quality video transmission services to users. Grid computing technology is a type of distributed parallel computing that can be used as a single supercomputer by utilizing the resources of multiple user PCs connected to the network. This grid computing technology is primarily used by platforms that provide peer-to-peer (P2P)-based services. Grid computing technology, which is a method of sharing internal resources between users, requires strict security control because data are exchanged without server intervention after a connection is established between a sender and receiver.

In this study, we analyzed the technical security vulnerabilities of grid computing, a technology that has been researched and developed since the early 2000s. A three-step analysis framework was developed and used to analyze a live-streaming platform that uses grid computing technology. The goal was to preemptively remove potential vulnerabilities by replacing them. Although grid computing technology is continuously developed and used, it does not utilize appropriate security verification, which may put users at risk. Previous studies have presented the architectural vision of grid computing, emphasized security, or reported security factors that should be considered in the P2P data communication of live-streaming services. However, research has not been conducted to prove the presence of these vulnerabilities by analyzing the data flow, creating a data flow diagram, identifying threats, and constructing attack scenarios. To address this, we propose a three-step vulnerability discovery framework. These three steps consist of a data flow analysis, threat modeling, and vulnerability analysis. An experiment environment was configured to control the access of service users, and a local experiment environment for a direct attack was established. Next, a data flow diagram (DFD) was derived by analyzing the flow of data based on an analysis of the process characteristics and protocols. Then, STRIDE threat modeling techniques were applied to identify threats and construct an attack tree and attack scenarios. The system vulnerability was verified by building it.

Our contributions can be summarized as follows:

- We provide a vulnerability discovery framework that can effectively analyze grid-computing-based client software;
- We analyze the vulnerabilities of actual services, which were not performed in previous studies, by using our proposed analysis framework. Several zero-day vulnerabilities were derived, thereby verifying our proposed analysis framework;
- We find four types of zero-day vulnerabilities, namely video stealing, information disclosure, denial of service, and remote code execution, which were derived by analyzing a live-streaming platform in Korea.

This paper provides details of the vulnerability analysis of a grid-computing-based live-streaming service and is structured as follows. Section 2 presents a review of related studies, the technology trends and security trends of grid computing systems, and the technical status of live-streaming services, the operational structure of live-streaming platforms in Korea, and threat modeling. Section 3 describes the three-step vulnerability discovery framework proposed for the data flow analysis and threat modeling of grid-computing-based applications. It includes the data flow analysis, threat modeling, and analysis of vulnerabilities. Section 4 shows the derivation of the zero-day vulnerability using the framework proposed for a grid-computing-based live-streaming service. Finally, Section 5 concludes this study.

## 2. Related Work

### 2.1. Grid Computing

Grid computing is an infrastructure that provides high computing capacity to distributed systems using widely distributed resources. The resources in grid computing are managed through the providers’ policies, calculations, frameworks, and cost and access models. Although the growth rate of the computing field is accelerating, owing to the availability of better hardware and software, the engineering and business fields require more effective handling because of the demand for grid computing. In the case of a service with a large quantity of data and numerous numerical calculations, the resources of a single device are limited, and grid computing is a suitable solution to this problem. Therefore, this section describes the technology trends of grid computing systems, as well as security trends.

#### 2.1.1. Technology Trends in Grid Computing Systems

**Figure 2:**
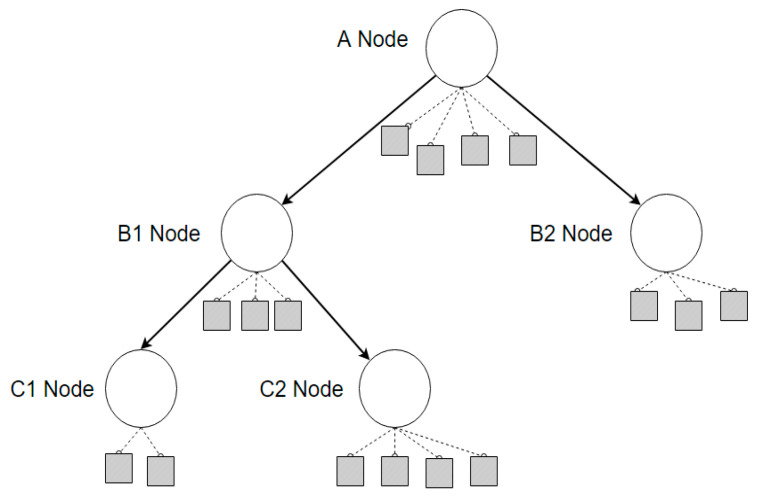
Grid computing method in a tree overlay network structure.

In tree-structured grid computing methods, a user receives data from a parent node and transmits it to a child node. The overall structure is such that data are transferred only in this direction, as shown in Figure 2. The most important feature of the tree overlay grid computing method is that only one path connects any two nodes. Therefore, it has the advantage of reduced computation compared to mesh overlay grid computing, and the use of a tree overlay structure has the greatest advantage when calculating properties using the shortest path. However, from a security point of view, if the user of the parent node modifies and transmits data to the lower child nodes, all child nodes connected to the parent node receive the tampered data. Because this can work efficiently, it requires robust data integrity verification.

**Figure 3:**
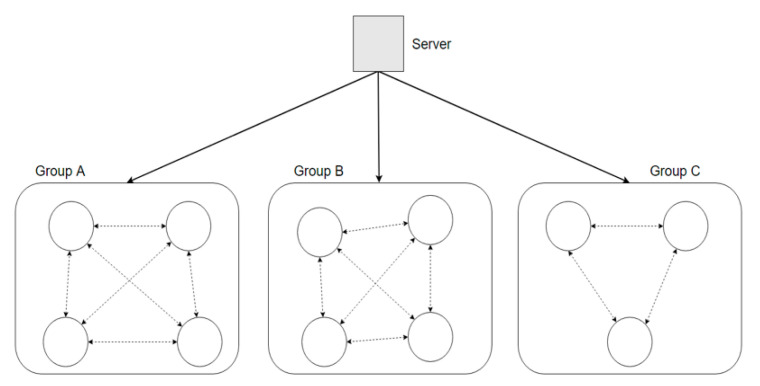
Grid computing method in a mesh overlay network structure.

In the mesh-structured grid computing method, data are transmitted and received by different users connected to the same group. As shown in Figure 3, this method is distinguished from the tree-structure grid computing method in which the parent node transmits data in one direction. Thus, when data are transmitted and received by users in the same group instead of one node, the risk of the tree-structured grid computing method is reduced. However, attacking users may be within the same group through data tampering.

#### 2.1.2. Research Trends on Grid Computing System Security

This section describes the security research trend for grid computing systems (Table 1). Seven studies were conducted in three main fields, each divided into security overview, risks, and security by design.

Grid computing is essentially an infrastructure that provides high computational capacity to distributed systems using widely, geographically distributed resources. As reported in Reference [2], although the hardware and network performance have improved over the past 15 years and the growth in computing has been good, problems in science, engineering, and business areas must be addressed more effectively. Therefore, this paper explains the need for grid computing technology for industries that require numerous resources because of the huge amount of numerical calculation and data processing. It also provides a discussion on different approaches for using distributed resources in a grid computing manner, such as cluster, cloud, meta-, and distributed computing manners.

In Reference [3], the security requirements and solutions in grid and cloud computing environments were investigated. Security problems were classified according to authentication, access control, integrity, confidentiality, and multiple security, and comparative studies were performed between the different technologies presented in each environment class. The latest technologies were used to solve security problems that can occur in both grid and cloud computing environments. These were categorized by assigning them to the areas of authentication, access control, integrity, and confidentiality. The proposed methods for solving specific problems in a grid environment were compared based on several important criteria. Access control was identified as the most important factor in both environments. In Reference [4], the use of a verification technique such as model checking was proposed to verify the security requirements of a grid system.

As stated in Reference [5], grid computing can assist in overcoming heterogeneity in terms of computing elements, operating systems, policymaking, and environment, but security issues hinder the adoption of grids as a broad IT virtualization solution. Thus, solutions to address these issues must be developed, and the following steps should be taken.

1. Identify specific vulnerabilities, threats, and policy issues in the current grid implementation.
2. Develop a realistic threat model based on the identified threats.
3. Develop countermeasures based on the developed threat model.
4. Perform quantitative and qualitative evaluations of the developed solution.

This approach was argued to enable the development of more complementary solutions and provide valuable insight into the nature of security attacks, thereby enabling the continuous development of better solutions.

Grid computing was overviewed in Reference [6]. Chapter 7 (“Security of Part 2. Grid architecture considerations”) is noteworthy. This chapter describes the security issues, techniques, and solutions required to provide a secure grid computing environment. In particular, five requirements are discussed: authentication, access control, data integrity and confidentiality, and key management.

1. Authentication: This is the process of validating the claimed individual and identifying the user. It is not limited to users, as it also refers to the authentication of services, applications, and other entities.
2. Access Control: This refers to ensuring each user or computer using the service can perform the requested action.
3. Data Integrity: This factor prevents unauthorized changes to data.
4. Data Confidentiality: This factor ensures sensitive information is not disclosed to unintended users and is often referred to as privacy.
5. Key Management: This refers to the secure creation, distribution, authentication, and storage of keys used for encryption. Based on the above factors, it describes the technology managed by the server and client when implementing a grid computing system.

In Reference [7], the forms of grid computing used worldwide were investigated. Based on this, the operation of grid software and its roles were explained using the grid software Globus Toolkit as an example. In particular, the necessary components of the grid software were emphasized and explained to allow the grid to be easily utilized in industrial applications. Lead computing consists of heterogeneous computers and resources distributed across multiple administrative domains to provide users with uniform access to resources. Users can access resources in the grid in multiple manners, each with unique security requirements and implications for both resource users and providers. In Reference [8], a comprehensive grid usage scenario was presented with respect to security requirements, such as authentication, authorization, integrity, and confidentiality. These scenarios primarily intend to provide the application designer with a library that can be matched to the system, thereby facilitating the use and development of applications that consider security in the early stages of design and innovation.

This analysis of the security research trend for grid computing systems included seven earlier studies, which can be divided into three fields. We derived the research results with additional factors and explained the potential security factors that should be strengthened based on the scenario in the case of risk. Finally, for earlier studies from a design perspective, an analysis was conducted of the security issues to be considered when designing a system in the development stage. From a security perspective, this means prior studies have not been conducted on grid computing systems in service or on determining vulnerabilities based on a systematic analysis framework.

### 2.2. Live-Streaming Service

This section describes the technology status and security research trends of live-streaming services and the operation structure of live-streaming platforms in Korea using grid computing systems.

#### 2.2.1. Research Trends on Grid Computing System Security

**Figure 4:**
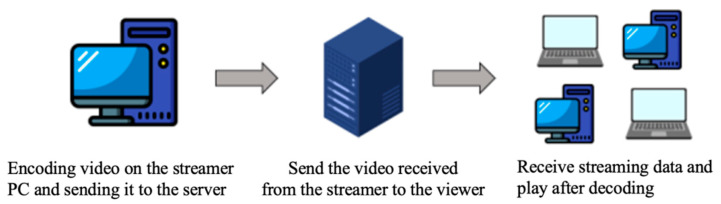
Streaming service data transmission path.

Owing to the recent developments in the media content industry, live-streaming services are changing from one-way communication to multi-way communication. In addition, the demand for a live-streaming platform with multi-directional communication is increasing with the number of Internet users and smart devices. A live-streaming service centrally manages an authentication server and streaming server to apply application layer multicast, and each user performs a streaming relay to other users. Here, the authentication server provides only an authentication mechanism for logging in through a web page, and the generated cookie information of users allows them to broadcast or watch broadcasts. In addition, as shown in Figure 4, a streaming server exists between the sender and the receiver, and it properly distributes the streaming data encoded in the PC of the sender to prevent the load from being concentrated by creating a multicast tree. It also optimizes and reconstructs trees. Such a server-based method has the advantage of a high creation speed because the tree is configured based on the server, but the possibility of a single point of failure still exists [9].

#### 2.2.2. Live-Streaming Platforms in Korea

In this study, as shown in Table 2, three live-streaming platform companies in Korea using grid computing technology were analyzed. These platforms exchange data in different manners depending on the operation structure of the grid computing system, but the structures of the process operations in the local environment are similar.

**Figure 5:**
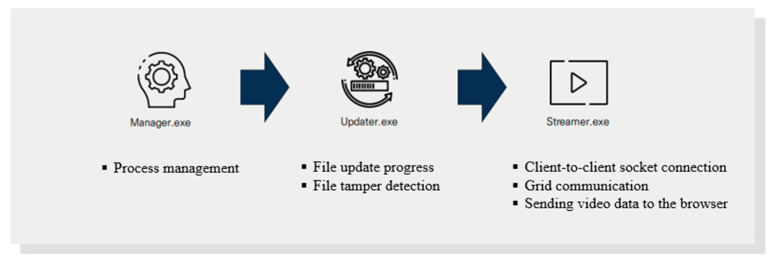
Client process flow.

For grid computing, three processes are in the local environment of the live-streaming platform, as shown in Figure 5. The Manager process is responsible for the overall management of the three processes. The Updater process checks for file tampering and updates files. Finally, the Streamer process handles socket connections between clients and transmits image data to the browser for grid communication.

**Figure 6:**
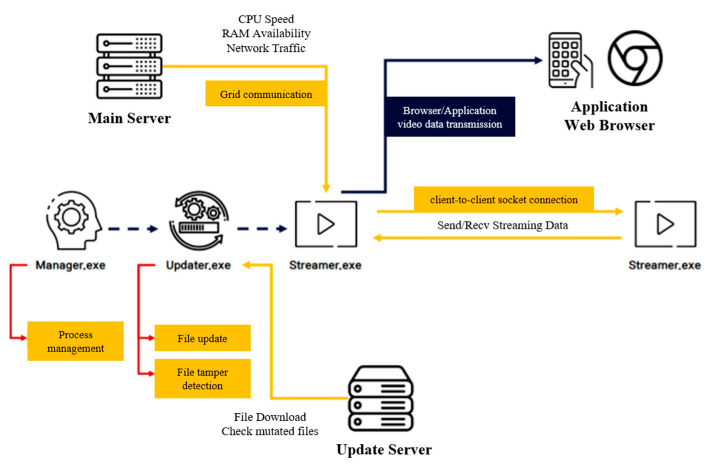
Grid computing operation structure in live-streaming services.

Figure 6 presents the operation structure for the grid computing of a live-streaming platform. When the user uses the Manger process of the live-streaming service, the Update process is executed by employing the ShellExecute() API. The Update process communicates with the updated server to obtain information about the latest file and checks whether the file has been forged by verifying the signature on the file. Subsequently, the Streamer process is executed. It exchanges CPU and RAM availability information with the main server, and, in grid computing, it determines whether the node has sufficient resources as the parent node attempts a socket connection. Thereafter, streaming data are transmitted/received from the connected node, and video data are transmitted to the browser and application to allow users watching the corresponding channel to view the received video.

#### 2.2.3. Security Research Trends

Three research topics can be identified in the security research trend for live-streaming services. These studies focused on the security threats that can occur when using P2P. The researchers in [10] proposed that, although research on the security of P2P streaming has begun to be actively conducted, a comprehensive security analysis of the current P2P solution has not been conducted. The best practices are not outlined in studies on system design, widely accepted attack models, measurement-based security threats in P2P streaming, and the examination of specific security aspects of these systems. Therefore, the study investigated these aspects and divided the types of attacks into Forgery, Pollution, Eclipse, Neighbor, Sybil, DoS, and Omission. Subsequently, it explained the security threats that can occur in tree- and mesh-based P2P streaming services and emphasized that user authentication, such as access control, is required.

In Reference [11], the problems established in published studies were reviewed and approaches for potential solutions to protect P2P-based voice and video streaming applications were outlined.

1. Authentication: Related studies have determined that fake nodes can be created. Therefore, authenticating the user ID is an additional task, and an appropriate mechanism must exist for this.
2. Live communication availability requirements: Live communication applications require low latency and a high constant bandwidth for video. Because of these characteristics, real-time communication (RTC) applications are more vulnerable to availability attacks than other P2P applications. An attacker can severely degrade services by dropping or delaying messages sent over a P2P network.

As argued in Reference [12], the decentralized nature of P2P live systems makes them vulnerable to several types of attacks, of which contamination is the most detrimental. The use of multimedia stream authentication can help detect contaminated content and identify malicious users, but at a high cost. To this end, we propose a signature mechanism for application in P2P live streaming. This is the result of a study comparing overhead and security as evaluation factors.

These three studies indicated common security threats that can occur through data integrity, access control, and P2P communication and argued that DoS and content contamination attack these systems. Therefore, a live-streaming platform, which was the target of the vulnerability analysis in this study, can be expected to have the same attack vector.

### 2.3. Threat Modeling Methodology

In this study, we investigated a three-step analysis framework for grid-computing-based application vulnerability analysis, modeled threats to identify them prior to a vulnerability analysis, and created scenarios for each threat to analyze the vulnerabilities. This section describes the types of threat modeling from a related study, along with Microsoft’s STRIDE threat modeling, which was adopted and used in this study.

#### 2.3.1. Types of Threat Modeling

A typical threat modeling process includes threat intelligence, asset identification, and threat mapping. Each of these processes provides different insights and visibility into the security perimeter. The threat modeling methods include STRIDE, PASTA, Trike, and LINDDUN. Each of these provides a different manner of assessing threats faced by IT assets. Threat modeling involves developing tests and procedures to identify potential threats and respond accordingly. This includes understanding the impact of a threat on a system and classifying the threat.

1. STRIDE threat modeling: STRIDE is a threat model created by Microsoft to identify system threats. It is used with a target system model and is the most effective manner of evaluating individual systems [13,14,15].
2. Process for Attack Simulation and Threat Analysis (PASTA): PASTA is an attacker-centered methodology that consists of seven steps. It is designed to correlate business objectives with technical requirements, guiding organizations to dynamically identify, calculate, and prioritize threats [16].
3. Trike threat modeling: Trike is an integrated conceptual framework for security inspection from a risk management security perspective through threat model creation in a reliable and repeatable manner [17]. This is a threat modeling technique that identifies users and assets in the data and usage flows and derives risks to the asset by analyzing the user’s execution frequency for the four elements of the asset: Create, Read, Update, and Delete. Its features include identifying the vulnerabilities of assets using Attack Tree and Attack Library and managing assets from a risk management perspective.
4. LINDDUN Threat Modeling: LINDDUN addresses seven privacy-related threats [18,19]: linkability, identity, non-repudiation, detectability, information disclosure, unawareness, and non-compliance.

#### 2.3.2. Study on Vulnerability Analysis Using Threat Modeling

Table 3 lists studies in which vulnerabilities were derived by using threat modeling. Although the target of analysis is diverse, Microsoft’s STRIDE and LINDDUN were primarily used for threat modeling. If all vulnerabilities were not derived using STRIDE, we confirmed that another threat modeling method was used. We created a DFD to determine the analysis target and understand the data flow. Subsequently, all possible threats were derived using the threat modeling method. It creates an attack tree using common threats and identifies attacks that are likely to occur against the analysis target. Afterwards, a vulnerability analysis was performed to perform this attack.

Based on this, we determined that identifying security threats from a design perspective and applying STRIDE threat modeling that considers software vulnerabilities would be most appropriate for the analysis of vulnerabilities in this study, which considered a grid-computing-based live-streaming service. Therefore, we proposed an analysis framework that utilizes STRIDE’s data flow analysis and threat identification and created an attack tree based on the derived threats to finally compose an attack scenario.

#### 2.3.3. Microsoft STRIDE Threat Modeling

The types of threat modeling are diversely distributed. In this study, a STRIDE-based threat modeling methodology was adopted, and threats could be identified from software and network perspectives, as shown in Table 4, which was advantageous.

Microsoft proposed the STRIDE method, which considers the properties of six types of security threats, as listed in Table 4.

(1) Spoofing: legitimate user, process, or system element;
(2) Tampering: legitimate information modification and editing;
(3) Repudiation: refusal or denial of certain actions performed in the system;
(4) Information disclosure: data breach or unauthorized access to confidential information;
(5) Denial of Service: suspension of service for legitimate users;
(6) Elevation of Privilege: a user with limited privileges can access system elements with higher privileges.

STRIDE analyzes the vulnerabilities of each system component that an attacker can exploit to compromise an entire system, typically in three steps. The first step is to decompose the system into logical or structural components. A component may be an internal process element that communicates within the system, or an external element that communicates with the system. The next step is to draw a DFD to visualize the functions inside or outside the system. This DFD uses four standard symbols: External Entity (EE), Process (Process, P), Data Flow (DF), and Data Store (DS). Once the DFD is complete, the threat is identified through STRIDE. Subsequently, when a threat to each system component is identified, the vulnerability that causes the threat is investigated, and, as the final step, an effective mitigation strategy is established based on the discovered vulnerability.

## 3. Suggested Vulnerability Discovery Framework

### 3.1. Overview of Vulnerability Discovery Framework

**Figure 7:**
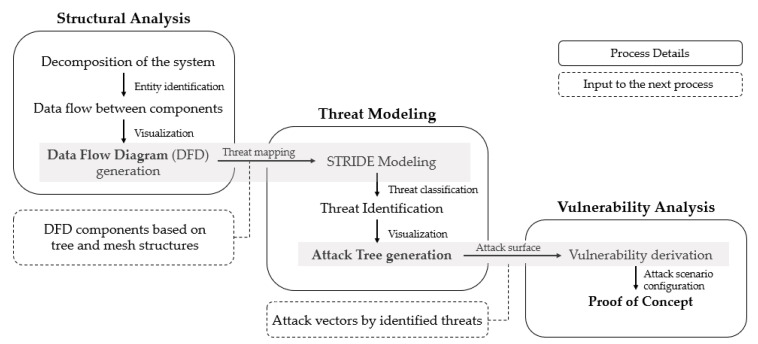
Vulnerability discovery framework composition.

To analyze the vulnerabilities of grid-computing-based live-streaming services, a framework for vulnerability detection was constructed, as shown in Figure 7. Naver TV, Kakao TV, and Afreeca TV, which utilize grid computing technology and are used for live streaming in Korea, were selected for analysis.

### 3.2. Structural Analysis

The structural analysis stage consisted of three detailed activities and was divided into the identification of major entities, identification of data flow between entities, and DFD creation. In this step, the framework was configured to identify major entities, such as processes and external objects, through an initial analysis and the data flow between entities through a network protocol analysis. The output was then structured by creating a data flow chart to visualize and identify threats. At this stage, processes, external objects, data flows, and trust boundaries were abstracted and could be expressed visually. The DFD was derived in this step and used as input for threat modeling.

### 3.3. Threat Modeling

In the third stage (the threat modeling stage), the framework was configured to perform three tasks. Based on the created data flow chart, STRIDE threat modeling was applied to identify threats, and the identified threats were analyzed to create an attack tree as the basis of the attack scenario. Thus, the threat identified in each entity was configured to allow its use in an attack scenario. At this stage, threats based on STRIDE were identified. The attack tree, which was the core of the threat modeling stage, was finally derived by analyzing the identified major threats. In the attack tree, the elements required for each attack were located based on the identified threats.

### 3.4. Vulnerability Analysis

In the vulnerability analysis stage, the attack tree constructed based on the previously derived threats was converted into an attack scenario that can be utilized in an actual grid computing system. Subsequently, the validity was determined by verifying the derived scenario to derive the zero-day vulnerability.

## 4. Experiments

This section describes the results of applying the proposed analysis framework for the vulnerability analysis of grid-computing-based live-streaming services, along with the results of establishing the environment configuration, data flow analysis, threat modeling, and vulnerability analysis.

### 4.1. Experimental Environment Configuration

On a live-streaming platform, establishing an analysis environment is essential to avoid a reduction in the availability of the service to its many users. To this end, an environment was configured such that the experiment could proceed without reducing the availability by implementing a “secret room” function provided by the live-streaming service. In addition, a P2P connection script was created to facilitate the connection between analysis PCs. Because of the nature of mesh-structured grid computing, two-way communication was required. Thus, connecting the analysis PCs to each other in a form different from a tree-structured experimental environment, which communicates in one direction, was necessary.

### 4.2. Results

This section describes the results of the experiment. The results of the data flow analysis are explained according to the grid computing structures of Naver TV, Kakao TV, and Afreeca TV, which were the analysis targets. Table 5 lists the DFD components.

#### 4.2.1. Data Flow Analysis in Tree-Structured Grid Computing Environments

**Figure 8:**
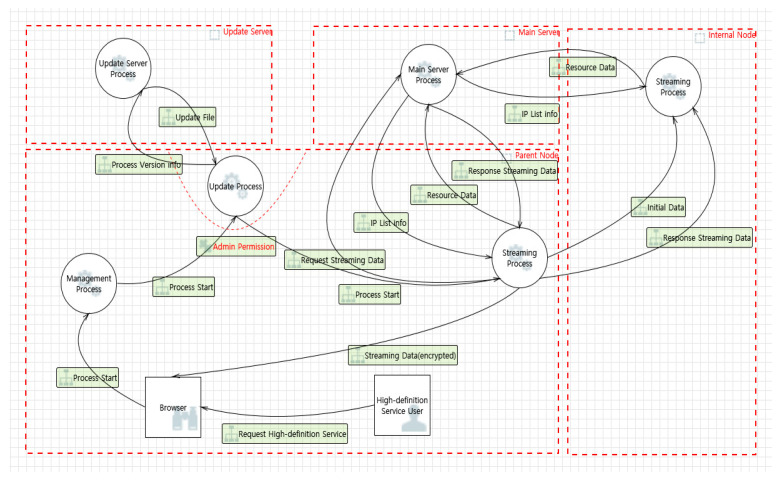
DFD of a tree-structure-based grid computing system.

Afreeca TV, which has a high usage rate in Korea, and Kakao TV, which has recently been in high demand because of its original content, use tree-structured grid computing systems. In tree-structure-based grid computing, as shown in Figure 8, five trust boundaries exist, along with two external entities, fifteen data flows, and six processes. Table A4 describes each component of the tree structure DFD.

#### 4.2.2. Data Flow Analysis in Mesh-Structured Grid Computing Environment

**Figure 9:**
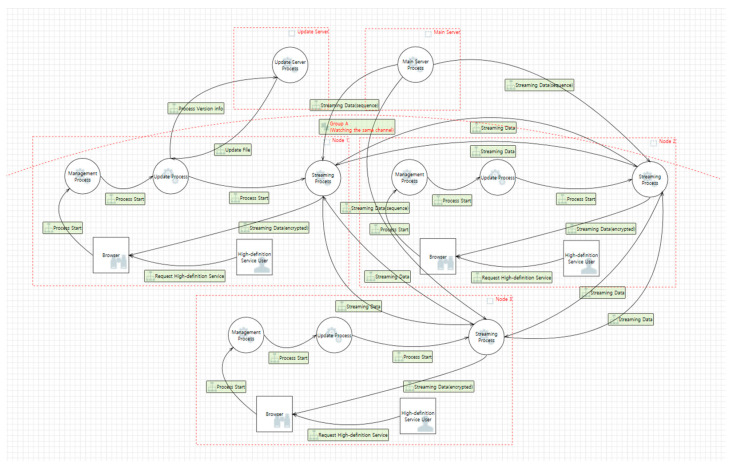
DFD of mesh-structure-based grid computing system.

Among the live-streaming services, Naver TV was the only service using the mesh structure, and the data flow chart prepared was configured accordingly, as shown in Figure 9.

The tree structure formed in grid computing differs significantly from a mesh structure because the streaming data received from the main server are managed in sequence and transmitted to the necessary nodes in the group. Therefore, streaming data containing sequences received from the Group A Trust Boundary and main server were added to the component. Table A5 describes each component of the mesh structure DFD.

#### 4.2.3. Threat Modeling Results

In this study, we used the STRIDE technique for threat modeling. This is a threat modeling technique proposed by Microsoft, which has six goals to achieve information protection for each element: the authentication, integrity, non-repudiation, confidentiality, availability, authorization, and identification of the symmetrical threats of spoofing, tampering, denial, information disclosure, denial of service, and elevation of privilege [19]. Table 6 lists the threats according to each component of the data flow chart based on the tree and mesh structure.

The tool used in this study was Microsoft’s Threat Modeling Tool Version 7.3.10801.1, which allows threats mapped to STRIDE to be automatically identified using the reporting function. Therefore, an analysis showed that the same types of threats existed in both the tree and mesh structures, with 134 and 133 threats derived from each, respectively. Thus, 267 threats were identified, which are too many to include in the main text. Table A1 in Appendix A lists 48 main threats.

**Figure 10:**
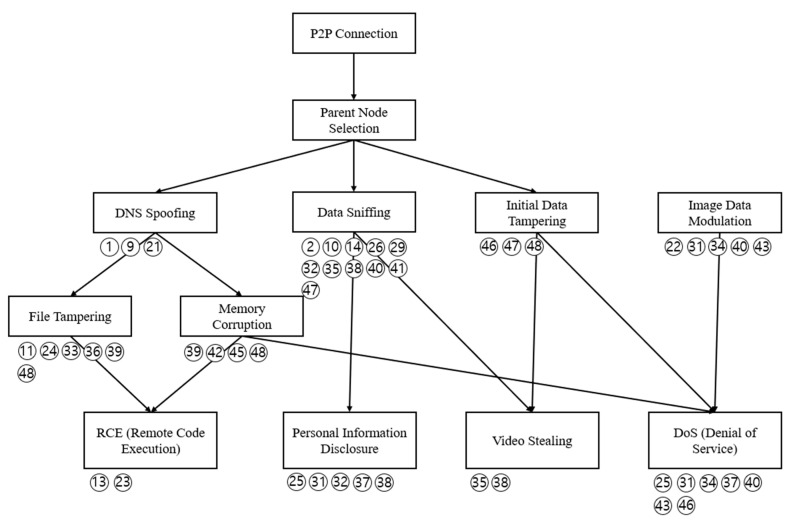
Derived attack tree.

Figure 10 shows an attack tree created based on the identified threats. A total of four attack trees were derived, each of which consisted of remote code execution (RCE), personal information disclosure, video stealing, and DoS.

#### 4.2.4. Attack Scenario Configuration

**Figure 11:**
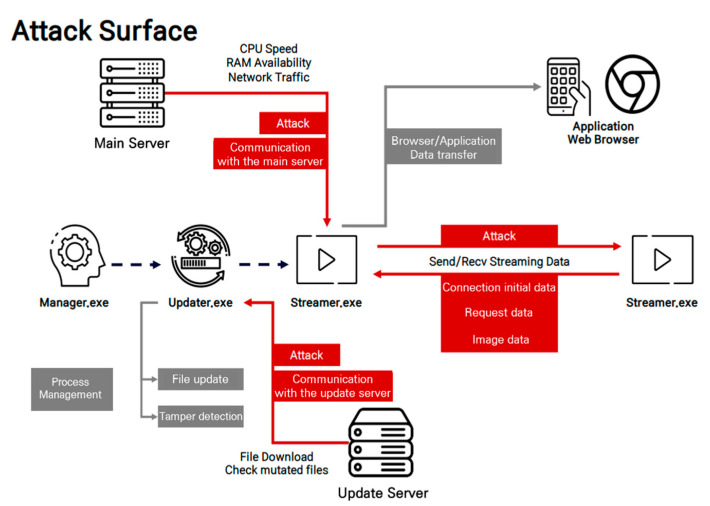
Attack surface discovered based on identified threats.

This section describes the attack surface discovered based on the threats derived above, and the attack scenarios that can be exploited. Figure 11 shows the attack surface discovered based on the identified threats. It comprises four attack vectors, each of which provides communication with the main server, communication with the update server, initial data in the client P2P connection process, video request data in the client P2P communication process, and video request data in the client P2P communication process. The attack scenarios included key information exposure during communication with the main server, update file tampering and remote code execution through DNS spoofing, image theft by data tampering at the beginning of a connection, DoS attacks through request-based index access, and pirate broadcasting through video data tampering.

The main server provides information about the node to be connected to in the grid computing processor transmitting the streaming data requested by the user. To this end, the streamer process delivers information about its CPU speed and RAM availability to the main server, and then the main server delivers information about the IP and port of the node connected to the streamer process. In this process, the possibility of exposing information on the IP list was confirmed, which occurred during grid computing with a mesh structure, and was analyzed as unnecessary work such that the private IP of the group was transmitted to form a group. Therefore, because a private IP can specify an individual, specifying a user watching a specific channel is possible when abused. The update server communicates with the local updater process. This confirms that the updater requests the file version from the server to obtain the latest file information, and the server uses the HTTP protocol. Therefore, in the LAN environment, an RCE attack is expected to be possible by tampering with the DNS.

The initial data of the client P2P connection process is connected to sub-nodes or grouped nodes to share streaming data in both tree- and mesh-structured grid computing. To this end, the initial data are used to identify each other between streamer clients. If a mechanism does not exist to authenticate this, attacks such as memory corruption and image stealing are expected through data tampering. When a P2P connection is established between clients through the previous initial data, in a grid computing environment with a mesh structure, a group is formed, and the sender and receiver broadcast the sequence number of the image data possessed by each client without distinction to request the necessary data. In this process, if the corresponding sequence number is tampered, the attacker can receive the video data of the desired frame or cause memory corruption. Thus, a DoS attack can occur.

In the grid computing used by live-streaming platforms, most of the packets are video data. However, if these are shared between sub-nodes and groups through grid computing communication, they can operate similar to a network worm even if the tampering is confined to only one data stream. Thus, maintaining integrity is an important factor. However, the analysis confirmed that the logic to verify the integrity does not exist, and if this is abused, pirate broadcasting can occur, allowing a user to watch unauthorized images through image data modulation, with the expectation that the largest part of the data exchanged would be available. Memory corruption is also an expected attack scenario.

#### 4.2.5. Proof-Of-Concept

**Figure 12:**
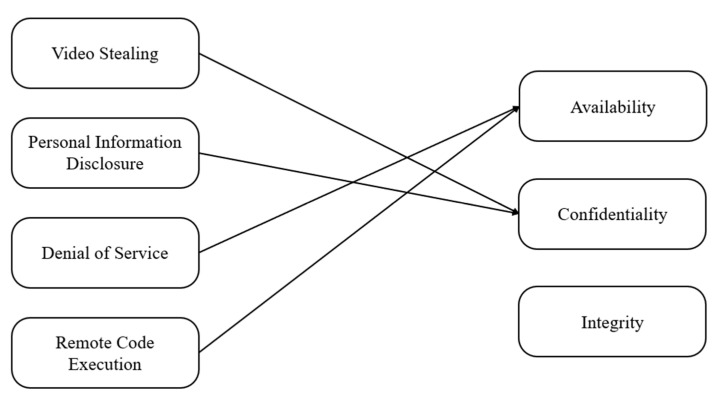
Security’s fundamental principles (CIA) that each attack violates.

This section describes the results of verifying the validity of the previously written scenario. Also, the tools used in the proof-of-concept experiment are described in detail in Appendix B. In this process, a number of vulnerabilities were derived, and depending on the configuration of the scenario, screen tampering, pirate broadcasting, and memory contamination vulnerabilities, including video stealing, personal information disclosure, and DoS and RCE attacks, were possible. As shown in Figure 12, video stealing and personal information disclosure threaten availability, while DoS and RCE threaten confidentiality.

- Video Stealing

**Figure A2:**
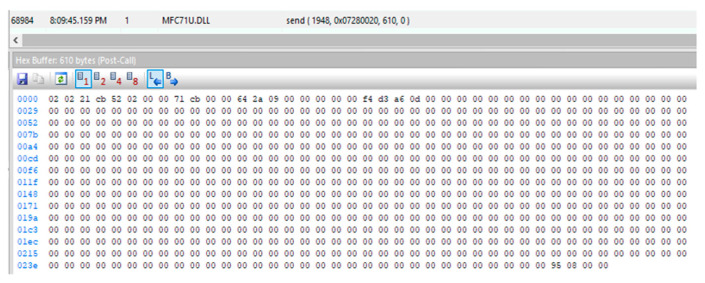
Initial data sent by the receiving client to the sending client.

**Figure A3:**
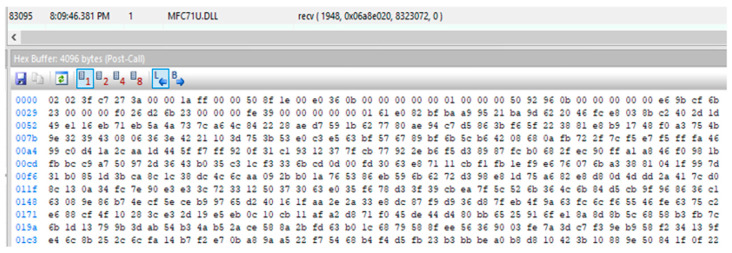
Process of receiving streaming data by connecting after sending all initial data.

**Figure A4:**
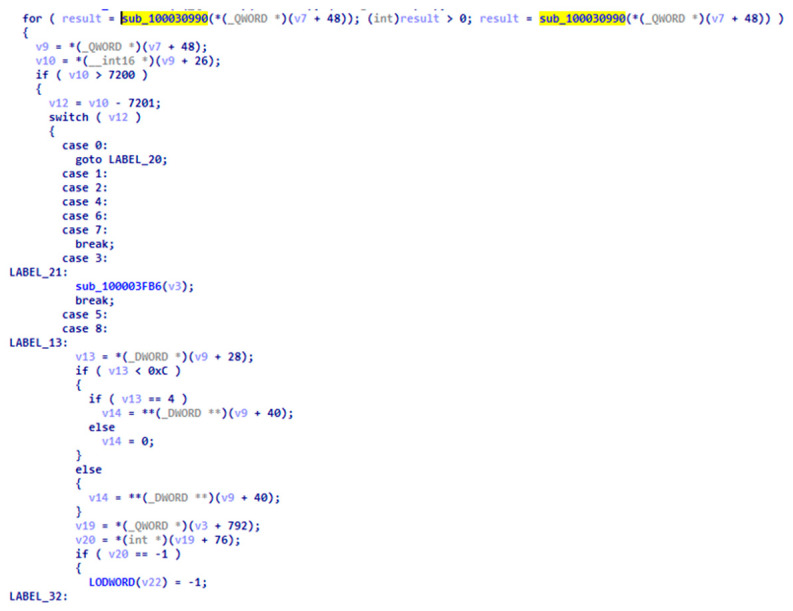
Data processing after calling recv().

Screen tampering vulnerabilities occurred when transmitting image data during P2P communication between clients. It is a vulnerability that occurred equally on all platforms. It is a serious vulnerability that leads to pirate broadcasting, which allows a receiver to control the screen being viewed on a specific platform. This occurs because of the absence of a routine for verifying the integrity of image data. Because the integrity is unverified, when the sender located in the parent node modulates data, the modulated image can be confirmed to have been transmitted to the child node and other nodes without filtering. After the receiving client sends the initial data to the sending client, the video and audio data receiving process occurs. At this time, the initial data are unverified. Thus, although the Streamer process is not running, it is a secret room without permission and age restrictions. We were able to steal images of rooms, etc. Figure A2 and Figure A3 show the memory dump results of the sending process of the initial data from the receiving client to the sending client and received streaming data. As shown in Figure A4, none of the processing or verification processes are performed after receiving the streaming data from the sender with the recv() function or before sending it to another node with the send() function.

- Personal Information Disclosure

**Figure A5:**
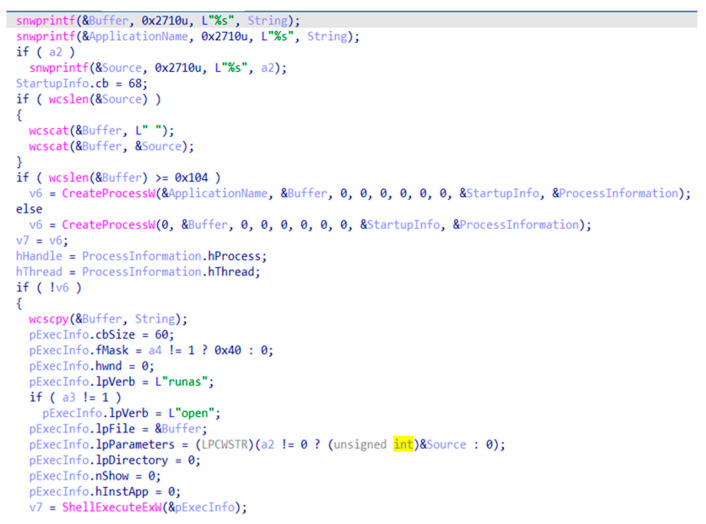
Code of process execution.

**Figure A6:**
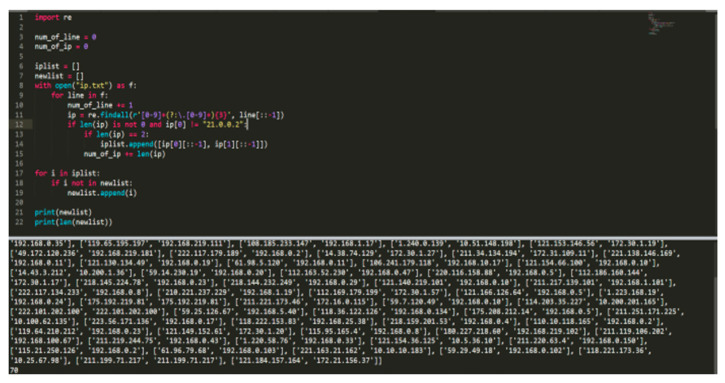
Verification script execution result.

A python script was written to steal data, and it proved that stealing video data from an unauthorized channel is possible by transmitting the initial data about the IP and targeted port. The code in Figure A5 is part of a function that executes a file in the Management process. The file name is received as an argument to the Buffer variable, and the Updater process is executed through the creatProcessW or ShellExecuteExW() function. Therefore, the address of the update server can be set as the address of the attacker server through DNS Spoofing, and malicious code can be executed on the user’s PC, watching the same channel in the LAN environment. This vulnerability occurred in the nodes constituting a group in the mesh structure. This was derived through a packet analysis using Wireshark and designed to receive private IP information when trying to connect to the same public IP band. In this case, because it can refer to a specific person, this can be said to expose personal information. As shown in Figure A6, a verification script is written to collect the private IP watching the channel, thereby proving the vulnerability.

- DoS and RCE

**Figure A7:**
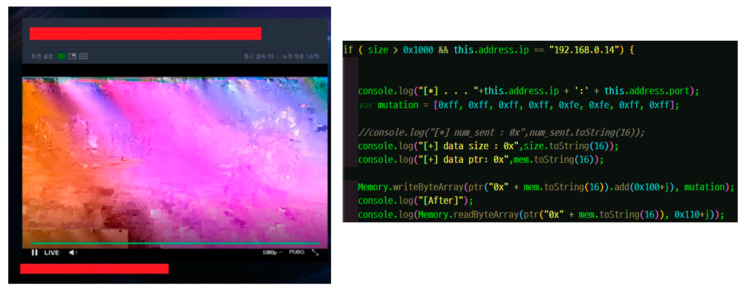
Example of DoS attack through video data tempering (picture distortion).

In the DoS attack depicted in Figure A7, among the attack vectors, the attack occurred during the initial data transmission process of the client P2P connection, image request data transmission, and image data transmission processes.

1. DoS attack through connection and initial data tampering: A packet analysis confirmed that the user was authenticated using the ticket value received from the server. In addition, if the ticket length value was altered to make it larger than the length defined in the ticket-related structure, the process was confirmed to terminate without exception, and the availability reduced. Therefore, this corresponded to a DoS attack.
2. DoS attack through request-based index access: The mesh-structured grid computing environment forms a group to transmit and receive data. As a result of modulating the sequence number according to the attack scenario configured, a crash was confirmed to occur outside the packet range, and the process terminated.
3. DoS attack through video data tampering: In the case of image data, a DoS attack was confirmed to be possible in the tree structure. When modulating the header and video data of the protocol, a field responsible for the length of the corresponding packet data existed.

If the vulnerabilities occurring in these three vectors are exploited, users cannot use high-definition services while using live-streaming services. In particular, DoS attacks caused by memory corruption were prevalent. This was classified as a fatal vulnerability because of the possibility of RCE. All vulnerabilities verified earlier occurred because grid computing was utilized and demand an improvement because they affect all connected nodes. Table 7 summarizes the problems that can occur through the vulnerabilities proven through proof of concept and the countermeasures to the vulnerabilities.

## 5. Conclusions

In this study, we analyzed the grid computing systems of live-streaming platforms that can be easily accessed in Korea to determine their technical security. To this end, a three-step vulnerability discovery framework was proposed. In an experiment, system threats were identified by configuring the experiment environment, data flow analysis, and STRIDE-based threat modeling, and an analysis was conducted to identify the vulnerabilities. The availability was secured by opening a secret room and using an automatic P2P connection script, and four attack scenarios were created using STRIDE and attack tree methods to identify the security threats in grid computing systems. After these were validated, deriving more than 10 zero-day vulnerabilities was possible in the grid-computing-based live-streaming services. The vulnerabilities identified can cause economic damage and leak users’ personal information. Moreover, when an attack occurs, these were proved to act similar to a network worm that affects an individual user and all nodes. Therefore, using a grid computing system requires authentication between users and data integrity verification. We analyzed the vulnerabilities of the most accessible live-streaming platform in Korea to determine the security in grid computing systems.

However, we did not analyze all services using different grid computing systems. Thus, our proposed vulnerability detection framework cannot be a standard of vulnerability detection for services using grid computing systems. To compensate for this, we are investigating services that use overseas grid computing systems. In future research, the proposed vulnerability detection framework will be validated for overseas services.

## Figures and Tables

**Table 1:** List of the studies on the research trend for grid computing system security.

	Paper Title	Year	Field
[2]	An Overview of Grid Computing	2019	(1) Security overview
[3]	Grid and Cloud Computing Security: A Comparative Survey	2019	(2) Risks, Access control
[4]	Model Checking Grid Security	2013	(3) Security by design
[5]	Grid Computing Security: A taxonomy	2008	(2) Risks, (3) Security by design
[6]	Introduction to Grid Computing	2005	(1) Security overview, (3) Security by design
[7]	A Gentle Introduction to Grid Computing and Technologies	2005	(1) Security overview
[8]	Security Implications of Typical Grid Computing Usage Scenarios	2002	(2) Risks, Scenario

**Table 2:** Live-streaming platforms in Korea using grid computing technology.

No.	Platform Name	Grid Computing Architecture	Main Content
1	Afreeca TV	Tree	One-person broadcast contents
2	Kakao TV	Tree	Original contents
3	Naver TV	Mesh	Sports

**Table 3:** List of studies in which vulnerabilities were derived by using threat modeling.

	Analysis Target	Year	Used Threat Modeling
[20]	Security Requirements of Smart Factory	2017	STRIDE
[21]	Security Requirements of Smart Home Hub	2018	LINDDUN
[22]	AI Speaker	2018	STRIDE, LINDDUN
[23]	Smart Speaker	2019	STRIDE
[24]	PS4 Remote Play with PC	2018	STRIDE
[25]	Smart Band	2018	STRIDE
[26]	Security Requirements of Electric Vehicle Charging Infrastructure	2017	STRIDE

**Table 4:** Attribute classification according to threats.

Threat	Desired Property
Spoofing	Authenticity
Tampering	Integrity
Repudiation	Non-repudiation
Information Disclosure	Confidentiality
Denial of Service	Availability
Elevation of Privilege	Authorization

**Table 5:** Components of DFD.

Components	Description	Figure
External Entity	External objects create data input and check output	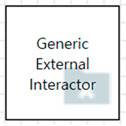
Data Store	Data stores store data temporarily or permanently	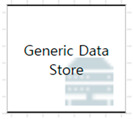
Process	Processes are responsible for taking data input and generating output	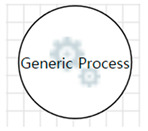
Data Flow	Data flow refers to the movement of data between objects	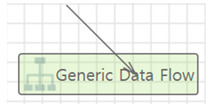
Trust Boundary	Trust boundaries represent changes in privilege levels	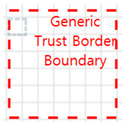

**Table 6:** Threats available in DFD components based on tree and mesh structures.

Threat	S	T	R	I	D	E
External Entity	✓					
Process	✓	✓	✓		✓	✓
Data Flow		✓		✓	✓	

**Table 7:** Issue caused by vulnerability and countermeasure to vulnerability.

Vulnerability	Issue Caused by Vulnerability	Countermeasure
Video Stealing	Stealing video data from unauthorized channels	An authentication process for the sender who sends data and encryption of the data sent by the sender are required. A procedure is required to verify the data that the sender node sends to the lower node through the checksum.
DoS	During the live-streaming service, the process is abnormally terminated, or the high-definition service cannot be used	
Personal Information Disclosure	Collect private IPs watching a specific channel	Deletes the logic of providing private IP during communication with the main server.
RCE	Executes malicious code on the user’s PC watching the same channel in a LAN environment	HTTPS (HTTP Secure) must be used so that DNS spoofing is not possible. A procedure for verifying local files is necessary by introducing file verification and signatures.

**Figure A1:**
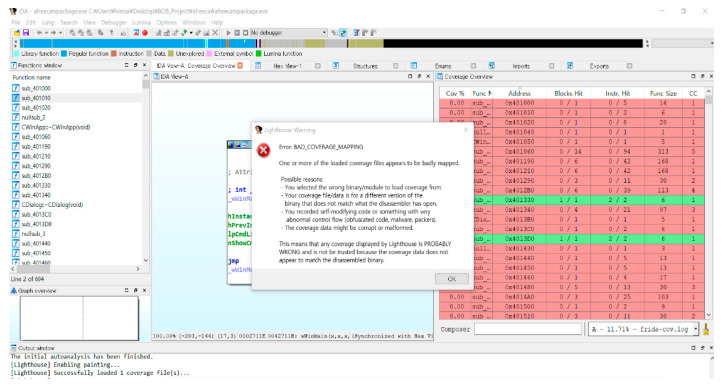
Example of DoS attack through video data tampering (picture distortion).

## Appendix A

**Table A1:** Main threats of grid-computing-based live-streaming services.

Element	Element Name	STRIDE	Threat Analysis	Threat Number
External Entity	Browser	S	The browser may be spoofed by an attacker, which may lead to unauthorized access to the Management Process. Consider using a standard authentication mechanism to identify the external entity.	T1
		E	Cross-site request forgery (CSRF or XSRF) is a type of attack in which an attacker forces a user’s browser to forge requests to a vulnerable site by exploiting an existing trusted relationship between the browser and vulnerable website.	T2
Process	Management Process	S	The Management Process may impersonate the context of the browser to obtain additional privilege.	T3
		E	The Management Process may impersonate the context of the browser to obtain additional privilege.	T4
	Update Process	S	The Update Process may be spoofed by an attacker, which may lead to unauthorized access to the Update Server Process. Consider using a standard authentication mechanism to identify the source/destination process.	T5
		R	The Update Process claims it did not receive data from a source outside the trust boundary. Consider using logging or auditing to record the source, time, and summary of the received data.	T6
		D	The Update Process crashes, halts, stops, or runs slowly, which violates an availability metric in all cases.	T7
		E	The Update Process may impersonate the context of the Management Process to obtain additional privilege.	T8
	Streaming Process	S	The Streaming Process may be spoofed by an attacker, which may lead to information disclosure through the Streaming Process. Consider using a standard authentication mechanism to identify the source/destination process.	T9
		R	The Streaming Process claims it did not receive data from a source outside the trust boundary. Consider using logging or auditing to record the source, time, and summary of the received data.	T10
		D	The Streaming Process crashes, halts, stops, or runs slowly, which violates an availability metric in all cases.	T11
		E	The Streaming Process may impersonate the context of the Update Process to obtain additional privilege.	T12
		E	The Streaming Process may remotely execute code for the Streaming Process.	T13
		E	An attacker may pass data to the Streaming Process to change the flow of program execution within the Streaming Process to the attacker’s choosing.	T14
	Update Server	S	The Update Server Process may be spoofed by an attacker, which may lead to information disclosure through the Update Process. Consider using a standard authentication mechanism to identify the source/destination process.	T15
		R	The Update Server Process claims it did not receive data from a source outside the trust boundary. Consider using logging or auditing to record the source, time, and summary of the received data.	T16
		D	The Update Server Process crashes, halts, stops, or runs slowly, which violates an availability metric in all cases.	T17
		E	The Update Server Process may impersonate the context of the Update Process to obtain additional privilege.	T18
		E	Update Process may be able to remotely execute code for Update Server Process.	T19
		E	An attacker may pass data to the Update Server Process to change the flow of program execution within the Update Server Process to the attacker’s choosing.	T20
	Main Server	S	The Main Server Process may be spoofed by an attacker, which may lead to unauthorized access through the Streaming Process. Consider using a standard authentication mechanism to identify the source/destination process.	T21
		E	The Streaming Process may impersonate the context of the Main Server Process to obtain additional privilege.	T22
		E	The Main Server Process may remotely execute code for the Streaming Process.	T23
		E	An attacker may pass data to the Streaming Process to change the flow of program execution within the Streaming Process to the attacker’s choosing.	T24
Data Flow	Update File	T	Data flowing through the Update File may be tampered by an attacker. This may lead to a denial-of-service attack against the Update Process, an elevation-of-privilege attack against the Update Process, or information disclosure through the Update Process.	T25
		I	Data flowing through the Update File may be sniffed by an attacker. Depending on the type of data an attacker can read, it may be used to attack other parts of the system or simply disclose information, which leads to compliance violations. Consider encrypting the data flow.	T26
		D	An external agent interrupts data flowing across a trust boundary in either direction.	T27
	Process Version Info	T	Data flowing across the Process Version Info may be tampered by an attacker. This may lead to a denial-of-service attack against the Update Server Process, an elevation-of-privilege attack against the Update Server Process, or information disclosure through the Update Server Process.	T28
		I	Data flowing across the Process Version info may be sniffed by an attacker. Depending on the type of data an attacker can read, it may be used to attack other parts of the system or simply disclose information, which leads to compliance violations. Consider encrypting the data flow.	T29
		D	An external agent interrupts data flowing across a trust boundary in either direction.	T30
	Streaming Data	T	Data flowing across the Streaming Data (sequence) may be tampered by an attacker. This may lead to a denial-of-service attack against the Streaming Process, an elevation-of-privilege attack against the Streaming Process, or information disclosure through the Streaming Process.	T31
		I	Data flowing across the Streaming Data (sequence) may be sniffed by an attacker. Depending on the type of data an attacker can read, it may be used to attack other parts of the system or simply disclose information, which leads to compliance violations. Consider encrypting the data flow.	T32
		D	An external agent interrupts data flowing across a trust boundary in either direction.	T33
	Resource Data	T	Data flowing across the Resource Data may be tampered by an attacker. This may lead to a denial-of-service attack against the Main Server Process, an elevation-of-privilege attack against the Main Server Process, or information disclosure through the Main Server Process.	T34
		I	Data flowing across the Resource Data may be sniffed by an attacker.	T35
		D	An external agent interrupts data flowing across a trust boundary in either direction.	T36
	IP List Info	T	Data flowing across the IP List Info may be tampered by an attacker. This may lead to a denial-of-service attack against the Streaming Process, an elevation-of-privilege attack against the Streaming Process, or information disclosure through the Streaming Process.	T37
		I	Data flowing across the IP List Info may be sniffed by an attacker. Depending on the type of data an attacker can read, it may be used to attack other parts of the system or simply disclose information, which leads to compliance violations.	T38
		D	An external agent interrupts data flowing across a trust boundary in either direction.	T39
	Request Streaming Data	T	Data flowing across the Request Streaming Data may be tampered by an attacker. This may lead to a denial-of-service attack against the Main Server Process, an elevation-of-privilege attack against the Main Server Process, or information disclosure through the Main Server Process.	T40
		I	Data flowing across the Request Streaming Data may be sniffed by an attacker. Depending on the type of data an attacker can read, it may be used to attack other parts of the system or simply disclose information, which leads to compliance violations. Consider encrypting the data flow.	T41
		D	An external agent interrupts data flowing across a trust boundary in either direction.	T42
	Response Streaming Data	T	Data flowing across the Response Streaming Data may be tampered by an attacker. This may lead to a denial-of-service attack against the Streaming Process, an elevation-of-privilege attack against Streaming Process, or information disclosure through the Streaming Process.	T43
		I	Data flowing across the Response Streaming Data may be sniffed by an attacker. Depending on the type of data an attacker can read, it may be used to attack other parts of the system or simply disclose information, which leads to compliance violations. Consider encrypting the data flow.	T44
		D	An external agent interrupts data flowing across a trust boundary in either direction.	T45
	Initial Data	T	Data flowing across the Initial Data may be tampered by an attacker. This may lead to a denial-of-service attack against the Streaming Process, an elevation-of-privilege attack against the Streaming Process, or information disclosure through the Streaming Process.	T46
		I	Data flowing across the Initial Data may be sniffed by an attacker. Depending on the type of data an attacker can read, it may be used to attack other parts of the system or simply disclose information, which leads to compliance violations.	T47
		D	An external agent interrupts data flowing across a trust boundary in either direction.	T48

## Appendix B. Tools Used in the Experiment

The tools used for vulnerability analysis are described below. Table A2 describes the tools used for the vulnerability analysis in the Proof-of-Concept. A disassembler was used for static code analysis, and tools such as windbg, x64dbg, and Cheat Engine were used for debugging. Subsequently, hooking was performed through frida for data modification, and Wireshark and API Monitor were used to analyze network traffic and APIs that process calls. Finally, lighthouse was used to check code coverage.

**Table A2:** List of tools used in the experiment.

Function	Tools	Description
Disassembler	IDA Pro	Disassembler for computer software
	Ghidra	Disassembler framework developed by the U.S. National Security Agency
	Binary Ninja	Reverse engineering platform developed by Vector 35 Inc
Debugging	windbg	Versatile debugger for Microsoft Windows
	x64dbg	Provides a UI (User Interface) that allows step-by-step debugging through the code running as an open-source debugger for Windows and check exactly what you are doing
	Cheat Engine	An open-source memory scanner and debugger primarily used for recompilation on Windows operating systems
Hooking	frida	Dynamic Binary Instrumentation (DBI) tool to run and analyze binaries dynamically with a dynamic instrumentation toolkit for developers and reverse engineering and security researchers
Network traffic analysis	wireshark	Used to analyze network traffic with an open-source packet analysis program
	API Monitor	Provides a function to monitor APIs called by programs written in C and C++ and check API call stacks, parameter values, and hex dumps.
Code coverage analysis	lighthouse	Code inspection explorer for IDA Pro and Binary Ninja, it can analyze the execution flow of code and provide it to analysts

### Appendix B.1. Disassembler

A disassembler is a computer program that converts machine language into assembly language. It is a tool that converts an analyst’s written program to be easy to read. In this experiment, it was used to analyze the executable files and DLL (Dynamic Link Library) of the software of the three companies analyzed.

### Appendix B.2. Debugging

A debugger is a computer program used to test and debug programs. In our experiment, to analyze the execution flow of the target program, we followed the input and output values and checked how the program operates according to the corresponding values.

### Appendix B.3. Hooking

Hooking refers to a command, method, technique, or action that intercepts function calls and events occurring between software components in various computer programs, such as operating systems and applications. In our experiment, because input values cannot be arbitrarily given owing to the nature of real-time streaming services, modifying the transmitted data is possible by hooking the send()/WSASend()or recv() APIs to prevent the crash that occurs when data is tampered in the local environment. Table A3 was written to modify the input value in the local environment by hooking the recv() API.

**Table A3:** Example of writing a hooking script using frida.

Process.enumerateModules(); var recv_address = Module.getExportByName('ws2_32.dll','recv'); var target_ip = ‘10.10.1.X’; var tmp = 1; Interceptor.attach(ptr(recv_address), { onEnter: function(args) { this.fd = args[0]; this.buf = args[1]; var address = Socket.peerAddress(parseInt(this.fd)); var header = Memory.readByteArray(ptr(parseInt(this.buf)), 0x4); var data = new Uint8Array(header); if ( address.ip == target_ip && this.len != 0x40000 && this.len >= 0x40 ) { console.log('========= onEnter ========='); console.log('buf : '+this.buf); console.log('len : '+this.len); console.log(Memory.readByteArray(ptr(parseInt(this.buf)), parseInt(0x40))); } }, onLeave: function(ret) { var address = Socket.peerAddress(parseInt(this.fd)); if ( address.ip == target_ip && this.len != 0x40000 && this.len >= 0x40 ) { console.log('========= onLeave ========='); console.log('buf : '+this.buf); console.log('len : '+this.len); // var mutaion = [0xad, 0xde, 0xef, 0xbe]; var mutaion = [0x41, 0x41]; var mutaion2 = [0x42, 0x42]; if( tmp == 1 ) { tmp = 0; Memory.writeByteArray(ptr(parseInt(this.buf)).add(0x35), mutaion); } else { tmp = 1; Memory.writeByteArray(ptr(parseInt(this.buf)).add(0x35), mutaion2); } console.log(Memory.readByteArray(ptr(parseInt(this.buf)), 0x40)); } } });

### Appendix B.4. Network Traffic Analysis

In the case of a real-time streaming service, data in a specific format is not transmitted, but a different protocol for each manufacturer is used. Therefore, in this experiment, tools such as Wireshark and API Monitor were used to understand the protocol structure and analyze it.

### Appendix B.5. Code Coverage Analysis

The code coverage was analyzed by modulating data through the hooking described in the application program analysis of the real-time streaming platform and then checking it. Representatively, lighthouse, a plug-in that can be used in IDA Pro and Binary Ninja, was used. As shown in Figure A1, lighthouse provides a function to indicate whether the code has been executed and check whether it has passed through the corresponding block. This was used to compare the process execution flow before data modification and the execution flow after modification, and it was used to derive vulnerabilities.

## Appendix C. Components of the DFD

**Table A4:** Components of a tree-structure-based grid computing system.

Group	Components	Description
External Entity	Browser	A medium that expresses encoded streaming data for users of streaming services
	High-definition Service User	Users who want to use high-quality streaming services
Process	Main Server Process	The server that selects a parent node based on the resource information of a user watching on a streaming channel and connects child nodes
	Update Server Process	The server where the latest files exist, and is in charge of version management of the three locally existing processes: Streaming, Update, and Management
	Management Process	The process responsible for running and managing a process that exists locally
	Update Process	When running, the process that communicates with the update server and receives the latest files
	Streaming Process	The process that shares data between nodes and transmits the received streaming data through a websocket connection to the browser
Data Flow	Process Start	Data used to execute a process using the ShellExecute() API
	Update File	Information about the latest file version since connecting to the update server
	Process Version Info	Version information of local files sent to the server
	Streaming Data (encrypted)	Streaming data encrypted using DTLS when delivered to the browser through a websocket connection
	Request High-definition Service	High-definition service request data that the user passes to the browser to receive high-definition streaming data
	Resource Data	Resource data transmitted to the main server during node selection process by checking the remaining amount of RAM and CPU
	Request Streaming Data	Streaming request data sent to the server or parent node when connecting to a channel
	Response Streaming Data	Response streaming data sent from the parent node or server to the child node in response to the streaming data request
	Initial Data	Initial data used for the initial connection between nodes
	IP List Info	Data about the IP to connect to, which the main server sends to the node
Trust Boundary	Update Server	The boundary where the update server is located
	Admin Permission	Boundary where processes with administrator privileges, instead of regular user privileges, are located in the local environment
	Parent Node	Parent node boundary that receives data from the server or parent node in the streaming process and transmits streaming data to lower nodes
	Main Server	Boundary where the main server is located
	Internal Node	Child node boundary that receives streaming data through the parent node during streaming

**Table A5:** Components of mesh-structure-based grid computing system.

Group	Components	Description
External Entity	Browser	A medium that expresses encoded streaming data to users of streaming services
	High-definition Service User	Users who want to use high-quality streaming services
Process	Main Server Process	The server that selects a parent node based on the resource information of a user watching a streaming channel and connects child nodes
	Update Server Process	The server where the latest files exist, and is in charge of version management of the three locally existing processes: Streaming, Update, and Management
	Management Process	The process responsible for running and managing a process that exists locally.
	Update Process	When running, the process that communicates with the update server and receives the latest files
	Streaming Process	The process that shares data between nodes and transmits the received streaming data through a websocket connection to the browser
Data Flow	Start Process	Data used to execute a process using the ShellExecute() API
	Update File	Information about the latest file version since connecting to the update server
	Process Version Info	Version information of local files sent to the server
	Streaming Data (encrypted)	Streaming data encrypted using DTLS when delivered to the browser through a websocket connection
	Request High-definition Service	High-definition service request data that the user passes to the browser to receive high-definition streaming data
	Streaming Data (sequence)	Streaming data with a sequence for sharing streaming data within a group
Trust Boundary	Update Server	The boundary where the update server is located
	Admin Permission	The boundary where processes with administrator privileges, instead of regular user privileges, are located in the local environment
	Nodes 1, 2, 3	The boundary where each connected node in the group is located
	Group A	The group boundary where each node is connected based on the IP information received from the main server for streaming data sharing
